# Paracrine IL-6 Signaling Confers Proliferation between Heterogeneous Inflammatory Breast Cancer Sub-Clones

**DOI:** 10.3390/cancers14092292

**Published:** 2022-05-04

**Authors:** Riley J. Morrow, Amr H. Allam, Belinda Yeo, Siddhartha Deb, Carmel Murone, Elgene Lim, Cameron N. Johnstone, Matthias Ernst

**Affiliations:** 1 Olivia Newton-John Cancer Research Institute, 145 Studley Rd, Heidelberg, VIC 3084, Australia; riley.morrow@onjcri.org.au (R.J.M.); amr.allam@onjcri.org.au (A.H.A.); belinda.yeo@onjcri.org.au (B.Y.); s.deb@anatpath.com.au (S.D.); car_mur@hotmail.com (C.M.); cameron.johnstone@onjcri.org.au (C.N.J.); 2 La Trobe University School of Cancer Medicine, 145 Studley Rd, Heidelberg, VIC 3084, Australia; 3 Department of Anatomical Pathology, Austin Hospital, 145 Studley Rd, Heidelberg, VIC 3084, Australia; 4 Garvan Institute of Medical Research, Darlinghurst, NSW 2010, Australia; e.lim@garvan.org.au; 5 School of Clinical Medicine, University of New South Wales, Randwick, NSW 2052, Australia; 6 Department of Clinical Pathology, University of Melbourne, Parkville, VIC 3010, Australia

**Keywords:** inflammatory breast cancer, HER2, pSTAT3, interleukin-6, heterogeneity, Tocilizumab, paracrine

## Abstract

**Simple Summary:**

This study provides novel mechanistic insights into the capacity of the inflammatory cytokine IL-6 and its associated STAT3-dependent signaling pathway to stimulate proliferation in trans between individual sub-clones in a model of heterogeneity in inflammatory breast cancer. The clinical relevance of this discovery is provided by our observation that proliferation of the IL-6 responsive subclone is sensitive to inhibition with the clinically approved anti-IL-6 receptor humanized monoclonal antibody Tocilizumab. These findings therefore provide a rationale for potentially repurposing Tocilizumab for the treatment of a subset of inflammatory breast cancer patients.

**Abstract:**

Inflammatory breast cancer (IBC) describes a highly aggressive form of breast cancer of diverse molecular subtypes and clonal heterogeneity across individual tumors. Accordingly, IBC is recognized by its clinical signs of inflammation, associated with expression of interleukin (IL)-6 and other inflammatory cytokines. Here, we investigate whether sub-clonal differences between expression of components of the IL-6 signaling cascade reveal a novel role for IL-6 to mediate a proliferative response in trans using two prototypical IBC cell lines. We find that SUM149 and SUM 190 cells faithfully replicate differential expression observed in a subset of human IBC specimens between IL-6, the activated form of the key downstream transcription factor STAT3, and of the HER2 receptor. Surprisingly, the high level of IL-6 produced by SUM149 cells activates STAT3 and stimulates proliferation in SUM190 cells, but not in SUM149 cells with low IL-6R expression. Importantly, SUM149 conditioned medium or co-culture with SUM149 cells induced growth of SUM190 cells, and this effect was abrogated by the IL-6R neutralizing antibody Tocilizumab. The results suggest a novel function for inter-clonal IL-6 signaling in IBC, whereby IL-6 promotes in trans proliferation of IL-6R and HER2-expressing responsive sub-clones and, therefore, may provide a vulnerability that can be exploited therapeutically by repurposing of a clinically approved antibody.

## 1. Introduction

Inflammatory breast cancer (IBC) is a relatively rare and highly aggressive form of breast adenocarcinoma that accounts for 8–10% of breast cancer-related mortality [1]. IBC is distinguished from the various molecular subtypes of non-IBC by its clinical presentation of inflammatory-like symptoms, including erythema, edema, tenderness, warmth, and dimpled skin [2]. IBC is diagnosed histologically by the invasion of tumor cells into the dermal lymphatic vessels or into the parenchyma of the mammary gland [3,4]. While IBC lesions may correspond to any of the major clinical subtypes of ductal adenocarcinoma (luminal A, luminal B, HER2 positive, or triple-negative (TNBC)), the more aggressive HER2-expressing and TNBC subtypes are over-represented among IBCs [5,6], with up to 40% of IBCs corresponding to the HER2 positive (HER2^pos^) subtype [7] and up to 30% designated as TNBC [8]. However, despite the introduction of multimodality treatment allowing for significant progress in past decades, survival rates of IBC patients remain a fraction of those with non-IBC [3,9].

Considerable efforts have been invested to identify inflammatory mediators as drivers of IBC, including the chemokines interleukin-8 (IL-8) [10] and C-C motif chemokine ligand-2 (CCL2)/monocyte chemoattractant protein-1 (MCP-1) [11]. Meanwhile, IL-6 produced by infiltrating macrophages, cancer associated fibroblasts, and other immune cells [12,13,14,15], is a recognized potent driver of tumor growth and metastatic progression across many solid malignancies, including breast cancer [14,16,17,18,19]. High levels of circulating IL-6 are associated with poorer survival of breast patients [12,20,21]. Indeed, over half of all primary human breast cancers and corresponding breast cancer cell lines show prominent accumulation of the activated form of the STAT3 transcription factor engaged by IL-6 signaling [17,22,23,24], suggesting widespread involvement of this pathway. Although additional intracellular pathways can be activated in response to IL-6 family cytokines [25], the cascade resulting in STAT3 activation is the most prominent and promotes a variety of both, tumor cell-intrinsic hallmarks of cancer as well as tumor-enabling cancer hallmarks conferred by cells of the cancer environment [26]. In primary IBC tumor specimens and corresponding cell lines excessive IL-6 expression is a recurring feature [24,27,28], which correlates with positivity for the activated and phosphorylated forms of JAK2 (pJAK2) and STAT3 (pSTAT3) [24].

Recent effort by Marusyk and others have started to provide insights into the various modes of interaction between individual clones within a given tumor as well as between tumor clones and stromal cells [29,30]. While such studies indicated frequent situations of clonal interference and competition, others resulted in mutual benefit, or synergy. Strikingly a majority of published studies on IBC assume, or at least imply, molecular homogeneity across the lesion [31,32,33], a concept that has clearly proven inadequate for many other types of solid malignancies, including the most common forms of breast cancer [34].

In this study, we sought to gain insight into the role of the IL-6 signaling pathway in IBC. The expression pattern of IL-6 and activity of the downstream effector STAT3 were evaluated by immunofluorescence in clinical IBC specimens and correlated with expression of the HER2 receptor. In a subset of tumors, co-localization of HER2 and activated STAT3 was evident in isolated tumor foci with IL-6 expressed in either adjacent tumor foci, adjacent stroma, or co-expressed with HER2 and pSTAT3. Using an in vitro complementation approach to model inter-clonal tumor cell interactions, we find that IL-6 secreted by HER2^neg^ and IL-6 unresponsive IBC cells drives proliferation of HER2^pos^ and IL-6R-expressing IBC cells. Importantly, this paracrine stimulation in trans is susceptible to inhibition with a clinically approved anti-IL-6R antibody. We therefore propose that IL-6 signaling may provide a therapeutic vulnerability arising from the clonal heterogeneity of IBC tumors to suppress growth and survival of the corresponding tumors.

## 2. Materials and Methods

### 2.1. Cell Lines and Cytokines

The HER2-positive IBC line SUM190 and the SUM149 IBC cell line corresponding to the TNBC subtype were obtained from Asterand Bioscience, Detroit, MI, USA (https://bioivt.com/, accessed on: 2 May 2022). FC-IBC-02 [35], BCX-010 [36], KPL4 [28] and MDA-IBC-3 [37] IBC cell lines were kindly provided by Naoto Ueno and Wendy Woodward (MD Anderson Cancer Centre, Houston, TX, USA). IBC cell lines were cultured in DMEM containing 10% fetal bovine serum (Moregate Biotech, Bulimba, QLD, Australia). MCF10A immortal human mammary epithelial cells were cultured as previously described [38]. Cells were maintained at 37 °C in a humidified atmosphere containing 5% CO_2_. Cell lines were tested regularly for the absence of mycoplasma. The identity of all breast cancer cell lines was authenticated by short tandem repeat (STR) profiling using the Gene Print 10 system (Promega Corporation, Alexandria, NSW, Australia) at the QIMR Berghofer Medical Research Institute (Herston, QLD, Australia). Recombinant human IL-6 (rhIL-6) was kindly provided by Richard Simpson (La Trobe University, Melbourne, Australia). For stimulation with rhIL-6, cells (2 × 10^5^ cells/well) were seeded into 6 well tissue culture plates unless otherwise stated. The following day cells were serum-starved for 24 h prior to stimulation with rhIL-6 in serum free medium (SFM) containing 0.1% BSA. Control cells were treated with SFM containing 0.1% BSA alone (vehicle). The anti-IL-6R antibody, Tocilizumab (Actemra^TM^) was obtained from Roche Pharmaceuticals (Hawthorn, VIC, Australia).

### 2.2. Quantitative Reverse Transcription-PCR (qRT-PCR)

Total RNA was isolated from cell lines using a High Pure RNA Isolation Kit (Roche Life Science, Merck Millipore, Bayswater, VIC, Australia). cDNA was synthesized from 1μg of total RNA using the High-Capacity cDNA Reverse Transcription Kit without RNase inhibitor (Thermo Fisher Scientific, Scoresby, VIC, Australia) in accordance with the manufacturer’s instructions. Gene expression was quantified by TaqMan qRT-PCR using Fast Universal PCR Mastermix (2×) no AmpErase UNG (Thermo Fisher Scientific) and a ViAA-7 real-time PCR instrument (Thermo Fisher Scientific). Expression of the gene of interest was normalized to expression of Ribosomal Protein L37A (RPL37A) using the comparative C*_T_* method [39], as previously described [40]. In certain experiments, the absolute expression level of the gene of interest was determined by linearizing the ΔC*_T_* value by computing 2^−^^ΔC^*^T^*. Assay IDs were: IL-6, Hs00174131_m1; IL-6R, Hs01075664_m1; IL6ST/GP130, Hs00174360_m1; ERB-B2 (HER2), Hs01001580_m1; SOCS3, Hs02330328_s1; RPL37A, Hs01102345_m1 (Thermo Fisher Scientific). Detection of alternatively spliced IL-6R encoding sIL-6R was carried out using a custom TaqMan assay, as previously described [41].

### 2.3. Detection of IL-6 Protein by ELISA

Cells were seeded at 2 × 10^5^ cells/well in 6-well plates and grown to approximately 80% confluence. Full-serum medium was then changed to SFM for 48 h and the conditioned medium collected, filtered, snap-frozen, and stored at −30 °C. Human IL-6 protein levels in conditioned medium were determined using a 96 well plate-based ELISA assay (Elisakit.com, Scoresby, VIC, Australia) in accordance with the manufacturer’s instructions. Data were normalized to total protein determined using the bicinchoninic acid (BCA) assay (Thermo Fisher Scientific) [42].

### 2.4. Western Blot Analysis and Quantification

Whole cell lysates were prepared using RIPA Lysis and Extraction Buffer (Thermo Fisher Scientific), supplemented with Phosphatase and Protease Inhibitor Cocktail tablets (Roche). NuPAGE LDS sample buffer and NuPAGE sample reducing agent (both from Thermo Fisher Scientific) were both added to protein samples according to the manufacturer’s instructions. Protein (30 μg) was separated by polyacrylamide gel electrophoresis using a Bis-Tris buffer system (Novex NuPAGE (4–12%), Thermo Fisher Scientific), prior to transfer to a 0.45 μm PVDF membrane (Immobilion-FL, Merck Millipore, Bayswater, VIC, Australia), and processed for fluorescence-based detection. The following primary antibodies and dilutions were used: pSTAT3 (Y705), 1:500 (Cell Signaling Technology, Danvers, MA, USA); total STAT3, 1:1000 (Cell Signaling Technology); GAPDH: 1:2000 (Sigma Aldrich, Castle Hill, NSW, Australia). The appropriate fluorescent secondary antibody (LI-COR Biosciences, Lincoln, NE, USA) was used and bands visualized and quantified using an Odyssey imaging system (LI-COR). Original western blots can be found at Figure S5.

### 2.5. Preparation of Concentrated Conditioned Medium from SUM149 Cells

SUM149 cells were grown to confluence in full serum media. Cells were washed thoroughly with PBS and switched to SFM/0.1% BSA for 24 h. Medium conditioned by subconfluent SUM149 cells was processed by filtering through a 0.22 μm polyethersulfone (PES) membrane (Merck Millipore) then concentrated by centrifugation using AmiconUltra-15 filter units with an Ultracel-10 membrane (Merck Millipore). An increase in concentration of 10–15 fold was obtained.

### 2.6. Enumeration of cell Number

SUM190 and SUM149 cells were seeded (4 × 10^3^ cells/well) into 96 well tissue culture plates in full serum medium. The following day, the medium was changed to SFM supplemented with 0.1% BSA. Cell density was determined 24 h later (d0) using the well imaging function on an Ensight Multimode Plate Reader (Perkin Elmer, Waltham, MA, USA). Cells were then incubated with combinations of rhIL-6, Tocilizumab and conditioned medium, or SFM/0.1% BSA alone (vehicle), and cell density was recorded every 24 h.

### 2.7. Breast Cancer Specimens and OPAL Multiplexed Multispectral Imaging for IL-6, HER2, and pSTAT3

All studies involving human tissues were approved by the Clinical Research Review Committee of Austin Health (CRRC). All formalin-fixed paraffin-embedded (FFPE) tissue from core-biopsied or surgically resected primary breast cancers were obtained from the Austin Hospital, Heidelberg, VIC, Australia, and sections were used at 4 μm. For heat-induced antigen retrieval, slides were incubated in an EDTA buffer (pH 9) at 95 °C for 20 min. Multiplex staining and imaging was conducted as previously described [43]. Each antibody in the panel was titrated for Opal^TM^ multiplex imaging before use in the full panel (Akoya Biosciences, Marlborough, MA, USA). Monoplex staining of each of the antibodies was performed using the Opal 7-color kit (Akoya Biosciences), and after the incubation with the appropriate HRP-conjugated secondary antibody, slides were gently incubated on a rocking platform with individual tyramide signal amplification (TSA)-conjugated fluorophores (Akoya Biosciences) for 5–10 min at room temperature, then washed three times in 1xTBST for 5 min. Glass cover slips were applied using aqueous mounting medium (Fluoromount-G, Thermo Fisher Scientific), and slides scanned at room temperature using a 20× objective and an automated quantitative pathology imaging system (Vectra^®^ 3, Akoya Biosciences). Using monoplex scans with optimal antibody concentrations, inForm software (Akoya Biosciences) was used to build a spectral library for the antigen of interest. Following optimization of staining parameters for each antibody, staining with the full multiplex panel was carried out (IL-6, HER2 and pSTAT3). The procedure for the first antibody on the multiplex panel (IL-6) was identical to that as for monoplex staining, except following addition of TSA dye and washing, antigen retrieval was carried out to remove antibody complexes. The procedure for the two subsequent antibodies (HER2 and then pSTAT3) was also identical to that for monoplex staining. Following addition of TSA dye and washing for the final antibody, slides were incubated with spectral DAPI (Akoya Biosciences) for 2.5 min with gentle rocking and then washed twice in water. Slides were cover slipped and scanned as described above. Images were spectrally dissected using InForm software with the previously established spectral library. The antibodies used were rabbit polyclonal anti-human IL-6 (Abcam, Melbourne, VIC, Australia); mouse monoclonal anti-human HER2, clone 3B5 (Merck Millipore); rabbit polyclonal anti-human pSTAT3 (Y705) (Cell Signaling Technology).

### 2.8. In Vitro Co-Culture of SUM190 and SUM149 Cells

SUM190 cells were labeled at 37 °C with the cell-permeable green fluorescent dye carboxyfluorescein diacetate succinimidyl ester (CFDA-SE, Thermo Fisher Scientific) at 3.5μM (d0). After washing, CFDA-SE^pos^/DRAQ7^neg^ viable cells were seeded (60,000 cells/well) into 48 well tissue culture plates in full medium (which is required for SUM190 cells to adhere), using a flow cytometer (FACSAria III, BD Biosciences, Scoresby, VIC, Australia) and incubated overnight. The following day the number of viable SUM190 cells (CFDA-SE^pos^/DRAQ7^neg^) was enumerated by flow cytometry in quadruplicate (FACS Canto II, BD Biosciences) to establish a baseline at d1. The remaining wells containing adherent SUM190 cells were then incubated with either low serum (0.25% FBS) alone, unlabeled viable SUM149 cells (90,000/well) in 0.25% FBS, or unlabeled viable SUM149 cells (90,000/well) in 0.25% FBS plus either the anti-IL-6R antibody Tocilizumab (Roche) or a control humanized IgG1. On d3 the total number of viable SUM190 cells (CFDA-SE^pos^/DRAQ7^neg^) was again enumerated by flow cytometry (FACS Canto II, BD Biosciences).

### 2.9. Statistical Analysis

Statistical analyzed were carried out using Prism v8 (GraphPad Software, San Diego, CA, USA) unless otherwise stated. *p* < 0.05 was taken as statistically significant.

## 3. Results

### 3.1. Heterogeneous STAT3 Activation in Human IBC Specimens

**Figure 1:**
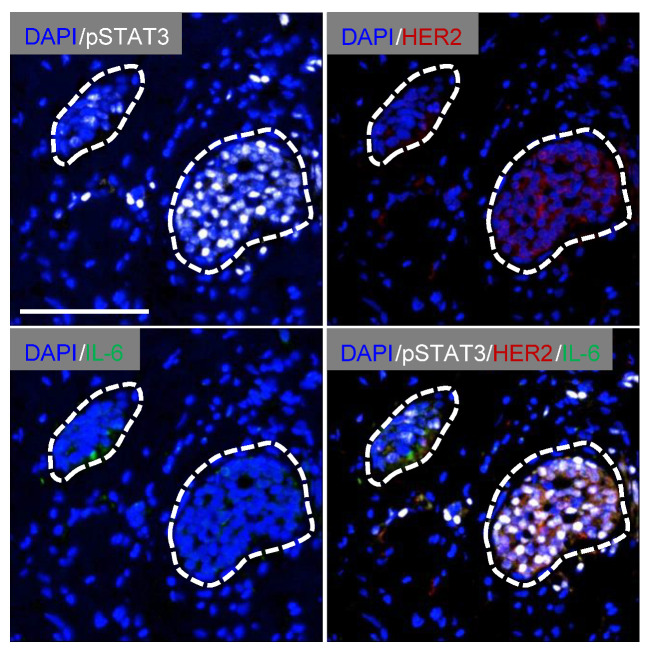
Localization of HER2, pSTAT3 and IL-6 in human inflammatory breast cancers. FFPE sections from human inflammatory breast cancer pre-treatment core biopsies were co-stained with HER2 (red), IL-6 (green) and pSTAT3 (white) antibodies using multiplexed multispectral imaging and counterstained with DAPI (blue) to visualize the nuclei (see Section 2). The specimen shown is a luminal A (ER^pos^, PgR^pos^, HER2^neg^) tumor (INFLAM #2, Table S1) which displays co-localization of cell surface HER2 expression and nuclear pSTAT3 in a cluster of tumor cells with diffuse IL-6 positivity in an adjacent tumor cell cluster. Scale bar represents 100 μm.

To investigate the IL-6 signaling pathway in human IBC, the distribution of IL-6, the tyrosine phosphorylated and activated form of the latent transcription factor STAT3 (pSTAT3) and HER2 protein was evaluated qualitatively in 15 formalin-fixed and paraffin-embedded core biopsy specimens of all clinical subtypes (luminal, HER2 amplified, and TNBC) from treatment-naïve IBC patients (Table S1). All patients presented with the classical clinical signs of IBC including skin erythema, and pathological evidence of dermal lymphatic involvement. The localization of IL-6, pSTAT3, and HER2 was determined using Opal multiplexed multi-spectral immunofluorescence analysis (see Section 2). Marked heterogeneity of expression of the three antigens was found with nine different patterns of expression revealed in IBC specimens. These are summarized in Table S1. Representative examples of three of the expression patterns (Pattern 2, Pattern 3, Pattern 4) are shown in Figure S1A–C, respectively. As expected, strong cell surface HER2 expression was found in the five tumors with *HER2* gene amplification, though both cell surface and cytoplasmic HER2 expression were also detected in tumors without HER2 amplification (INFLAM #2 (luminal), INFLAM #4 (luminal), INFLAM #6 (luminal), INFLAM #9 (TNBC), INFLAM #10 (TNBC), INFLAM #12 (luminal), Table S1). In tumors that stained positively for HER2 (HER2^pos^), cell surface HER2 expression in clusters of tumor cells was often co-localized with activated nuclear pSTAT3 (Table S1, Figure S1C), albeit with some heterogeneity of pSTAT3 staining, as described for other human breast tumor subtypes [17,22,23]. In tumors with co-expression of HER2 and pSTAT3, three patterns of IL-6 distribution were found. In the first instance, IL-6 immunoreactivity was observed in HER2^neg^/pSTAT3^neg^ tumor cell foci adjacent to clusters of HER2^pos^/pSTAT3^pos^ cells (Pattern 1, Figure 1). In the second, IL-6 was expressed in tumor stroma adjacent to regions with HER2^pos^/pSTAT3^pos^ tumor cells (Pattern 2, Figure S1A). Some nuclear pSTAT3 accumulation was also evident in the IL-6^pos^ regions of tumor stroma, suggesting potential autocrine IL-6 signaling in these areas. In the third pattern, HER2, pSTAT3, and IL-6 are all co-localized in tumor cell clusters (Pattern 4, Figure S1C), again suggesting potential autocrine IL-6 signaling in these regions. However, adjacent tumor regions with reciprocal expression of pSTAT3 and IL-6 was also observed in a HER2^neg^ tumor (Pattern 3, Figure S1B).

### 3.2. Expression of IL-6 Signaling Components in Human IBC-Derived Cancer Cell Lines

**Figure 2:**
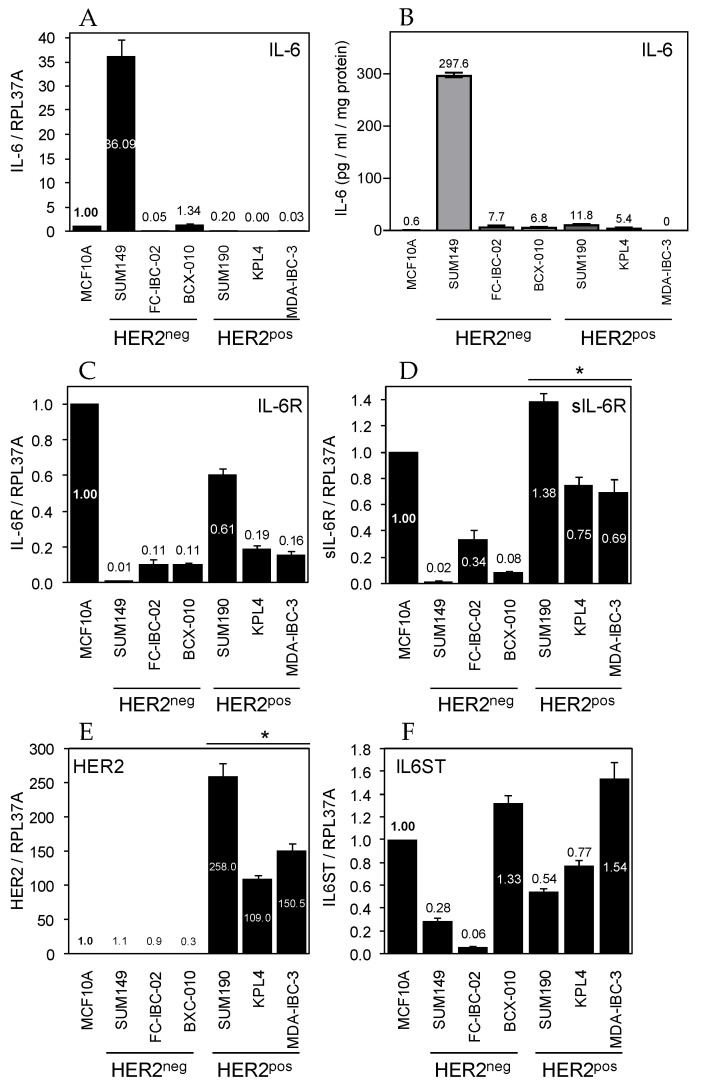
Expression of IL-6 signaling pathway components in human inflammatory breast cancer cell lines Basal mRNA levels of IL-6 (**A**), IL-6R (**C**), soluble IL-6R (**D**), HER2 (**E**), and IL6ST/GP130 (**F**) were evaluated by TaqMan qRT-PCR. Expression in immortal MCF10A mammary epithelial cells was set to 1. Mean ± SD (*n* = 3). The three HER2^neg^ and three HER2^pos^ cell lines are indicated. IL-6R and sIL-6R mRNA levels were highest in the HER2^pos^ cell lines. * *p* < 0.05. (**B**) Basal IL-6 protein levels in conditioned medium from the indicated cell lines were evaluated by ELISA. Mean ± SD (*n* = 4).

Given the multitude of factors capable of inducing STAT3 activity [26], we set out to explore a direct link between IL-6 and pSTAT3 in IBC. First, we evaluated the expression of IL-6 signaling components in the currently available *bona fide* human IBC-derived cell lines [5,28,35,36,37]. Six IBC cell lines were used for in vitro studies, three corresponding to TNBC cancers (SUM149, FC-IBC-02, BCX-010), which are HER2^neg^ and three were derived from HER2^pos^ cancers (SUM190, KPL4, MDA-IBC-3). Average IL-6 mRNA (Figure 2A) and secreted protein levels (Figure 2B) showed a trend for elevated expression in the HER2^neg^ lines compared to the HER2^pos^ lines, but differences did not reach statistical significance. While our finding of IL-6 expression in SUM149 cells concurred with previous publications [27,44,45], IL-6 protein expression in SUM149 was more than 50-fold higher than in the other HER2^neg^ cell lines (Figure 2B). This observation is consistent with findings in the recently described TNBC IBC cell line A3250, as interrogation of the associated RNA-Seq dataset revealed that cultured A3250 cells also synthesize excessive IL-6 mRNA, approximately four-fold higher than the levels in SUM149 cells (Figure S2). Interestingly, the A3250 line recapitulates in an orthotopic xenograft model some IBC-specific key features of the human disease including a biased recruitment of tumor associated macrophages and monocytes rather than granulocytes [11]. Conversely, mRNA levels encoding the full-length, membrane-bound IL-6 receptor alpha chains (IL-6R) were higher in HER2^pos^ lines than in HER2^neg^ lines (Figure 2C). Meanwhile, an alternatively spliced mRNA encoding a soluble isoform of IL-6R (sIL-6R) was expressed by all six IBC lines [46], consistent with previous reports [47]. Average levels of this isoform were significantly higher in HER2^pos^ lines than in the HER2^neg^ lines (Figure 2D). We also assessed expression of the shared IL-6 receptor beta chain IL6ST/GP130 which was expressed at high and similar levels across all six lines as expected (Figure 2F). The levels of HER2 mRNA in IBC cell lines (Figure 2E) correlated positively with both IL-6R and sIL-6R mRNA levels (Figure S3).

### 3.3. Differential Induction of pSTAT3 in Response to IL-6 in Human IBC-Derived Cancer Cell Lines

**Figure 3:**
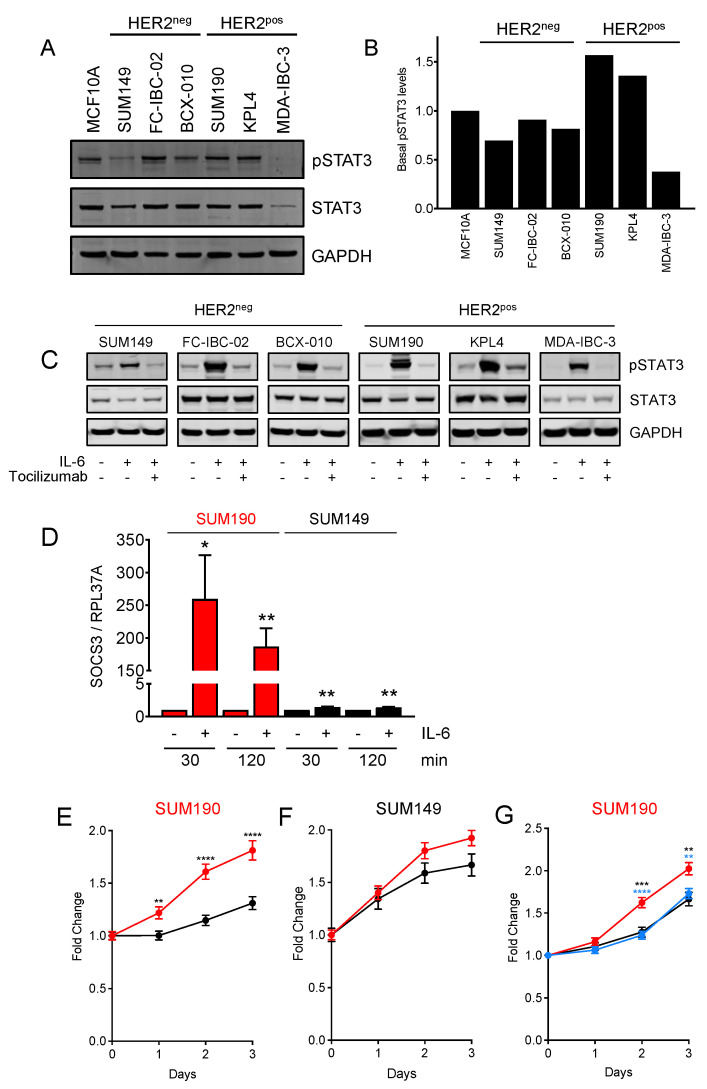
rhIL-6 induces STAT3 activation, induction of *SOCS3* target gene, and cell proliferation in SUM190 HER2^pos^ IBC cells. (**A**) Basal pSTAT3 (Y705) levels were evaluated in MCF10A cells and in IBC cell lines by Western blotting. Total STAT3 levels are also shown. GAPDH was used as a loading control. pSTAT3 was detected all six cell lines. (**B**) Quantification of pSTAT3 levels using densitometry. pSTAT3 levels were normalized to total STAT3 and GAPDH levels. pSTAT3 levels in MCF10A cells were set to 1. (**C**) rhIL-6 induced STAT3 activation was determined in IBC cell lines (three HER2^neg^, three HER2^pos^) by Western blotting. Cells were stimulated with vehicle or rhIL-6 (50 ng/mL) for 30 min or pre-treated with anti-IL-6R antibody Tocilizumab (50 µg/mL) for 1 h prior to rhIL-6 exposure (50 ng/mL, 30 min). Total STAT3 levels are also shown. GAPDH was used as a loading control. (**D**) SOCS3 mRNA levels were determined by TaqMan qRT-PCR in SUM190 cells or SUM149 cells following stimulation with rhIL-6 (50 ng/mL) or vehicle (-) for 30 or 120 min. Expression in vehicle treated cells was set to 1. Mean ± SD of 3 biological replicates is shown. * *p* < 0.05, ** *p* < 0.01. (**E**) SUM190 cell number was determined either in serum-free conditions (black line) or following exposure to rhIL-6 (red line, 50 ng/mL). ** *p* < 0.01, **** *p* < 0.0001. (**F**) SUM149 cell number was determined either in serum-free conditions (black line) or following exposure to rhIL-6 (red line, 50 ng/mL). (**G**) SUM190 cell number was determined either in serum-free conditions (black line), following exposure to rhIL-6 (red line, 50 ng/mL), or following Tocilizumab (50 µg/mL) plus rhIL-6 (50 ng/mL) exposure (blue line). Cell number was set to 1 at day 0. ** *p* < 0.01, *** *p* < 0.001, **** *p* < 0.0001. All proliferation assays shown mean ± SEM (*n* = 3). *p*-values were calculated using unpaired Student’s *t* test.

To determine the functional consequences of expression of the IL-6 ligand / receptor components described above, we assessed appearance of pSTAT3 as an unambiguous immediate intracellular signaling event triggered by ligand binding-induced formation of the IL-6;IL-6R;IL6ST/GP130 hexameric receptor complex and activation of the associated JAK family tyrosine kinases [26]. All IBC lines expressed STAT3 protein, albeit MDA-IBC-3 at lower level, and all but the MDA-IBC-3 line harbored also the activated pSTAT3 form when maintained in standard culture medium without specific stimulation (Figure 3A,B). The ability of each of the six IBC lines to specifically respond to IL-6 was then assessed by the induction of pSTAT3 in response to acute exposure to recombinant human IL-6 (rhIL-6). All cell lines responded to rhIL-6 stimulation with an increase in pSTAT3, which could be blocked by pre-incubation of the cultured cells with the neutralizing anti-IL-6R antibody Tocilizumab (Figure 3C). rhIL-6 mediated STAT3 activation was weakest in SUM149 cells, which correlated with low IL-6R expression (Figure 2C). Tocilizumab attenuated rhIL-6-induced pSTAT3 but not basal STAT3 activation as indicated by similar abundance of pSTAT3 in unstimulated and in IL-6-stimulated cells exposed to Tocilizumab (Figure 3C). We surmise from these observations that basal pSTAT3 activation in IBC cells under steady-state in vitro conditions occurs independently of IL-6 signaling.

### 3.4. Differential Proliferative Response to IL-6 in IBC Cell Lines

We next compared cellular responses to IL-6 between HER2^pos^ SUM190 cells harboring high levels of IL-6R expression and in HER2^neg^ SUM149 cells with low levels of IL-6R expression. The robust rhIL-6 induced STAT3 activation in SUM190 cells (Figure 3C) corresponded to a marked and transient induction of the STAT3 target gene *SOCS3* (Figure 3D). In comparison, the much less pronounced rhIL-6-dependent pSTAT3 induction in SUM149 cells correlated with a very weak *SOCS3* transcriptional response. Importantly, the differential activation of intracellular signaling was associated with a significant difference in the proliferative response of the two cell lines to rhIL-6 in serum free medium, with a significant increase in proliferation in SUM190 cells (Figure 3E), but not in SUM149 cells (Figure 3F). IL-6 was not induced in SUM190 cells when cultured in low serum or serum free conditions (Figure S4). IL-6 stimulated mitogenesis is rare in breast cancer cell lines, where IL-6 often has no effect or is anti-proliferative [48]. We conclude that the effect in SUM190 was due to the IL-6R-dependent signaling because Tocilizumab completely abrogated IL-6 induced proliferation in SUM190 cells (Figure 3G). Collectively, these data demonstrate that SUM190 cells respond to exogenous IL-6 with mitogenesis, which can be inhibited by IL-6R blockade with Tocilizumab.

### 3.5. IL-6 Confers a Proliferative Response in Trans across Individual IBC Cell Clones

**Figure 4:**
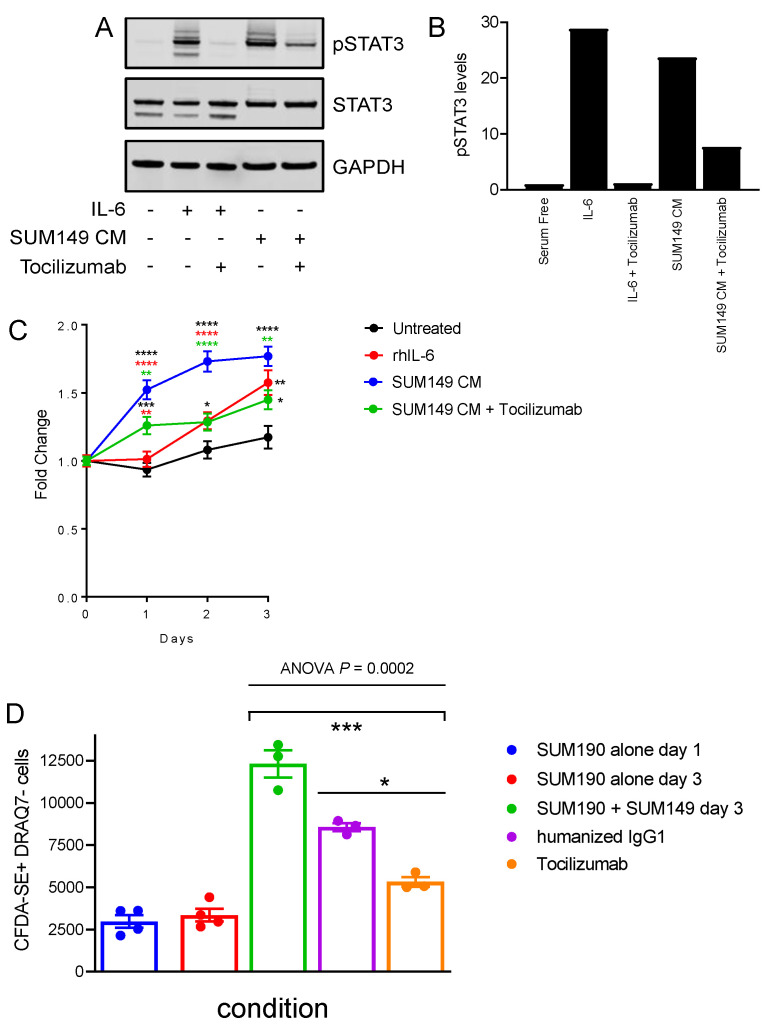
Endogenous IL-6 secreted from SUM149 cells stimulates STAT3 activation and proliferation in SUM190 HER2^pos^ IBC cells. (**A**) Cells were serum starved for 24 h and then pSTAT3 levels determined by Western blot in SUM190 cells that were exposed to rhIL-6 (50 ng/mL), conditioned medium from SUM149 cells, or vehicle (first lane) for 30 min. Prior to stimulation, some wells were incubated with Tocilizumab (50 μg/mL) for 1 h in serum free medium, as indicated. Total STAT3 levels are also shown. GAPDH was used as a loading control. (**B**) Quantification of pSTAT3 levels using densitometry. pSTAT3 levels were normalized to total STAT3 and GAPDH levels. pSTAT3 levels in cells serum starved for 24 h (serum free) was set to 1. (**C**) SUM190 cell number was determined in serum-free conditions (black line) or following exposure to rhIL-6 (red line, 50 ng/mL), concentrated conditioned medium from SUM149 cells (blue line) or concentrated conditioned medium from SUM149 cells plus Tocilizumab (green line, 50 µg/mL). The cell number at day 0 was set to 1. Mean ± SEM (*n* = 3). * *p* < 0.05, ** *p* < 0.01, *** *p* < 0.001 **** *p* < 0.0001. *P* values were calculated using unpaired Student’s *t* test. (**D**) CFDA-SE labeled SUM190 cells were cultured alone (*n* = 4) or co-cultured with an excess of unlabeled SUM149 cells (*n* = 3) over a 2 day period in the presence of 0.25% FBS, followed by determination of viable CFDA-SE+ cell number by flow cytometry. To determine the role of SUM149 cell-derived IL-6, Tocilizumab, or control humanized IgG1 were added to the co-cultures (2 μg/mL) at d1. Mean ± SEM. Data were analyzed by one-way ANOVA (*p* = 0.0002). Two-way comparisons were done using Tukey’s multiple comparisons test. Viable SUM190 cell numbers were significantly lower in cultures incubated with Tocilizumab compared to cultures incubated with the control humanized IgG1 (*, *p* < 0.05).

The observation of staining Patterns 1 and 3 in human IBC specimens (Figure 1, Figure S1B) raised the intriguing possibility that in certain IBC tumors, IL-6 may be supplied *in trans* from one tumor cell clone to a second clone expressing IL-6R. To explore whether IL-6 could mediate inter-clonal communication within a heterogeneous IBC tumor in vitro, we investigated whether supernatant from high IL-6-producing SUM149 cells could induce activation of the JAK-STAT3 signaling pathway and downstream effects in IL-6R positive SUM190 cells. Serum-free medium conditioned by SUM149 cells produced robust induction of pSTAT3 in SUM190 cells. We functionally confirmed that this activity was most likely due to IL-6, as pre-incubation of SUM190 cells with Tocilizumab abrogated most of the pSTAT3 induction conferred by conditioned medium (Figure 4A,B). We next compared exogenous rhIL-6 with SUM149 conditioned medium in terms of their ability to induce SUM190 cell proliferation (Figure 4C). SUM149 conditioned medium produced a superior mitogenic effect on SUM190 cells to 50 ng/mL rhIL-6 over a 3 day period. Importantly, a substantial fraction of the proliferative response conferred by SUM149 conditioned medium could be blocked by co-incubation with Tocilizumab (Figure 4C). This result showed that a sizeable proportion of the total mitogenic activity of SUM149 conditioned medium toward SUM190 cells is due to an activity that is neutralized by Tocilizumab (i.e., rhIL-6), though other factors capable of activating STAT3 are likely to also contribute.

### 3.6. In Vitro Complementation of IL-6-Mediated Inter-Clonal Stimulation of Proliferation

Finally, we sought to validate this finding using a low serum co-culture system between the two IBC cell lines that did not require collection and concentration of conditioned medium. For this, we labeled SUM190 cells a with fluorescent dye prior to seeding and then cultured them with or without addition of unlabeled SUM149 cells prior to the enumeration of viable fluorescent cells 2 days later. SUM190 cells alone showed a negligible increase in growth (Figure 4D). However, the addition of SUM149 cells increased the number of SUM190 cells by approximately four-fold. Again, we confirmed that the bulk of the induced SUM190 cell proliferation observed in co-cultures could be attributed to activation in trans by IL-6, because addition of Tocilizumab suppressed induction of SUM190 cell proliferation significantly more than an isotype-matched control antibody. Importantly, Tocilizumab reduced SUM190 proliferation in co-culture not below the negligible growth of SUM190 cells grown in monocultures, suggesting that Tocilizumab specifically inhibited activity resulting from the presence of SUM149 cells in the co-cultures. Collectively, these results provide evidence for inter-clonal growth support through a soluble factor rather than through physical cell-cell interaction.

## 4. Discussion

Both cellular clonal heterogeneity and variation in levels of target gene expression are forms of intra-tumoral heterogeneity that impair durable therapeutic responses in breast cancer [34,49]. Indeed, the presence of HER2 negative tumor cells in tumors classified clinically as HER2 positive and treated with HER2 targeting antibodies such as Trastuzumab is associated with de novo resistance to treatment and poor patient outcome [50,51,52]. In our analysis of HER2, IL-6, and pSTAT3 levels in a panel of primary IBCs, we found substantial co-localization of HER2 and pSTAT3, consistent with pSTAT3 as a known key regulator of growth in HER2^pos^ breast cancer cells [53,54]. However, several tumors displayed variation in expression of both cell surface HER2 and pSTAT3 in the tumor compartment, also consistent with reports in HER2 positive breast cancer [50,51,52]. Multiplexed co-immunofluorescence for all three antigens revealed distinct patterns of expression. In one pattern, HER2^pos^/pSTAT3^pos^/IL-6^neg^ tumor cell foci were identified adjacent to clusters that were HER2^neg^/pSTAT3^neg^/IL-6^pos^. Interestingly, this pattern was identified in a luminal tumor that was negative HER2 amplification. In a second pattern, the HER2^pos^/pSTAT3^pos^/IL-6^neg^ tumor cell clusters were adjacent to regions of stroma that were IL-6 positive. It is well established that IL-6 is produced by tumor infiltrating macrophages and other myeloid cells in both IBC [12,55,56] and non-IBC [14,17,57] specimens and by other cells in the tumor microenvironment such as cancer-associated fibroblasts [58]. Indeed, IBCs are enriched for tumor infiltrating macrophages relative to non-IBCs [10,11], and this feature has even been proposed to be one of the drivers of the unique IBC clinical phenotype [11]. However, our observation of adjacent pSTAT3^pos^/IL-6^neg^ and pSTAT3^neg^/IL-6^pos^ foci in a HER2^neg^ tumor, suggesting that there may not be an absolute (functional) correlation between HER2 expression and activation of STAT3 in transformed mammary epithelium. 

Based on the first and third scenarios, we hypothesized that certain clones within a tumor lesion may provide IL-6 *in trans* to promote the growth of adjacent clones. Thus, we tested this paracrine signaling hypothesis by examining human IBC derived cell lines. Firstly, expression of IL-6 signaling components was determined in a panel of human IBC cell lines. Expression of IL-6R mRNA was higher in the three HER2^pos^ lines, consistent with previous reports showing moderate HER2-induced upregulation of IL-6R expression [53,54]. In contrast, IL-6 mRNA levels were relatively lower in the three HER2^pos^ lines. Ectopic overexpression of HER2 strongly induced IL-6 expression in primary human mammary epithelial cells, MCF10A immortal mammary epithelial cells, luminal MCF-7 cells and 4T1 mouse TNBC cells [53,54,59]. However, concordant with our data, steady state IL-6 levels in HER2^pos^ breast cancer cell lines were low [59]. Korkaya and colleagues demonstrated that IL-6 was induced in HER2^pos^ breast cancer cells by the HER2 neutralizing antibody Trastuzumab in the setting of *PTEN* loss, with *PTEN* inactivation being a known mechanism of resistance to HER2-directed therapies [59,60]. Interestingly, SUM149 cells are *PTEN*^null^, which might drive the strong upregulation of IL-6 seen in this line [61].

Here we experimentally test the hypothesis that intra-tumoral heterogeneity with respect to STAT3 activity, as indicated by the appearance of tyrosine 705 phosphorylated STAT3, may provide a molecular mechanism whereby individual clones of transformed cells may cooperate to promote and sustain IBC progression. Surprisingly, we find endogenous production of IL-6 by SUM149 IBC cells that express low levels of IL-6R, and hence remain resistant to stimulation with the ligand, can provide this cytokine in trans to stimulate the growth of the IL-6 responsive and IL-6R positive cell line SUM190. The findings here expand the concept of commensal paracrine growth support by IL-6 over and above that of stromal-derived IL-6 from M2 polarized tumor-associated macrophages and mesenchymal stem cells [12,13]. Thus, this discovery is significant in light of observations that tumor-associated macrophages and monocytes are not only more abundant in IBC than in non-IBC specimens [11,24,62], but also provide a source for inflammatory cytokines when exposed to the IBC cell-derived chemokine CCL2 [63]. Although the IL-6 “trans-signaling” phenomenon provides a mechanism to confer IL-6 responsiveness to cells with insufficient IL-6R expression by inflammation-associated shedding of the extracellular region of IL-6R from neutrophils [64], IBC lesions show remarkably low levels of intratumoral neutrophils [4,11]. Thus, neutrophil-mediated IL-6 trans-signaling is unlikely to “restore” IL-6 responsiveness to HER2^neg^/IL-6R^low^ SUM149-like tumor cells.

Interestingly, the IL6ST/GP130-JAK-STAT3 signaling cascade was recently identified as a non-cell-autonomous driver of tumor growth in a xenograft model addressing inter-clonal interactions in TNBC [30]. Activation of this signaling cascade was critical to retain the overall heterogeneity of the tumor, and equilibrium among all clones was required for sustained tumor growth. Furthermore, interference with the IL6ST/GP130-JAK-STAT3 signaling cascade resulted in induction of necrotic collapse leading to a failure of tumor growth. Strikingly, retention of clonal heterogeneity was dependent on clonal expression of IL-11, the most closely related cytokine to IL-6. These observations are reminiscent of the findings here that co-culture of two IBC cell lines can confer a selective growth advantage to one cell line but not the other. Therapeutic targeting of the IL-6-IL6ST/GP130-JAK-STAT cascade in breast cancer is currently being explored in a phase I clinical trial using the anti-IL-6R antibody Tocilizumab in combination with HER2-targeting agents in patients with HER2-positive tumors resistant to first-line therapy with Trastuzumab (ClinicalTrials.gov Identifier: NCT03135171). Meanwhile, Tocilizumab has been approved as first-line treatment for Castleman’s disease and systemic juvenile idiopathic arthritis [60]. Consistent with our findings, Tocilizumab also reduced proliferation of SUM190 cells when cultured as three-dimensional organoids in vitro [47].

## 5. Conclusions

Our data showed that SUM149-derived IL-6 confers growth stimulation to SUM190 cells through a Tocilizumab-sensitive mechanism and suggests that this paracrine effect may sustain a long-term growth-promoting equilibrium between two such tumor cell clones in vivo. The data provide a rationale to explore repurposing of Tocilizumab and other clinically approved monoclonal antibodies interfering with binding of IL-6 to its cognate receptor subunit to test their clinical efficacy for the treatment for IBC patients with clinical evidence of IL-6-dependent STAT3 activation in their lesions.

## Data Availability

The data presented in this study are available within the manuscript and in the Supplementary Materials Data files.

## Supplementary Materials

The following supporting information can be downloaded at: https://www.mdpi.com/article/10.3390/cancers14092292/s1, Figure S1: Localization of HER2, pSTAT3 and IL-6 in human inflammatory breast cancers by OPAL/Vectra staining; Figure S2: RNA-Seq analysis of IL-6 mRNA expression in cultured TNBC cell lines; Figure S3: Correlation between IL-6 and HER2 mRNA expression in IBC cell lines; Figure S4: TaqMan qRT-PCR analysis of IL-6 (black) and IL-6R (orange) mRNA expression in SUM190 cells in full serum (10%) and low serum conditions; Figure S5: Original Western blots corresponding to Figure 3A,C and Figure 4A; Table S1: Clinical variables of the human inflammatory breast cancer cohort.
