# Supplementary material for: Paracrine IL-6 Signaling Confers Proliferation between Heterogeneous Inflammatory Breast Cancer Sub-Clones
Source: Cancers (Basel). 2022 May 4;14(9):2292. doi: 10.3390/cancers14092292 (PMC9105876; doi:10.3390/cancers14092292)
Supplement: Supplementary file 1 [file cancers-14-02292-s001.zip › cancers-1599349-suppl.pdf]

Supplementary Materials: Paracrine IL-6 Signaling Confers Proliferation between Heterogeneous Inflammatory Breast Cancer Sub-Clones

Riley J. Morrow, Amr H. Allam, Belinda Yeo, Siddhartha Deb, Carmel Murone, Elgene Lim, Cameron N. Johnstone and Matthias Ernst

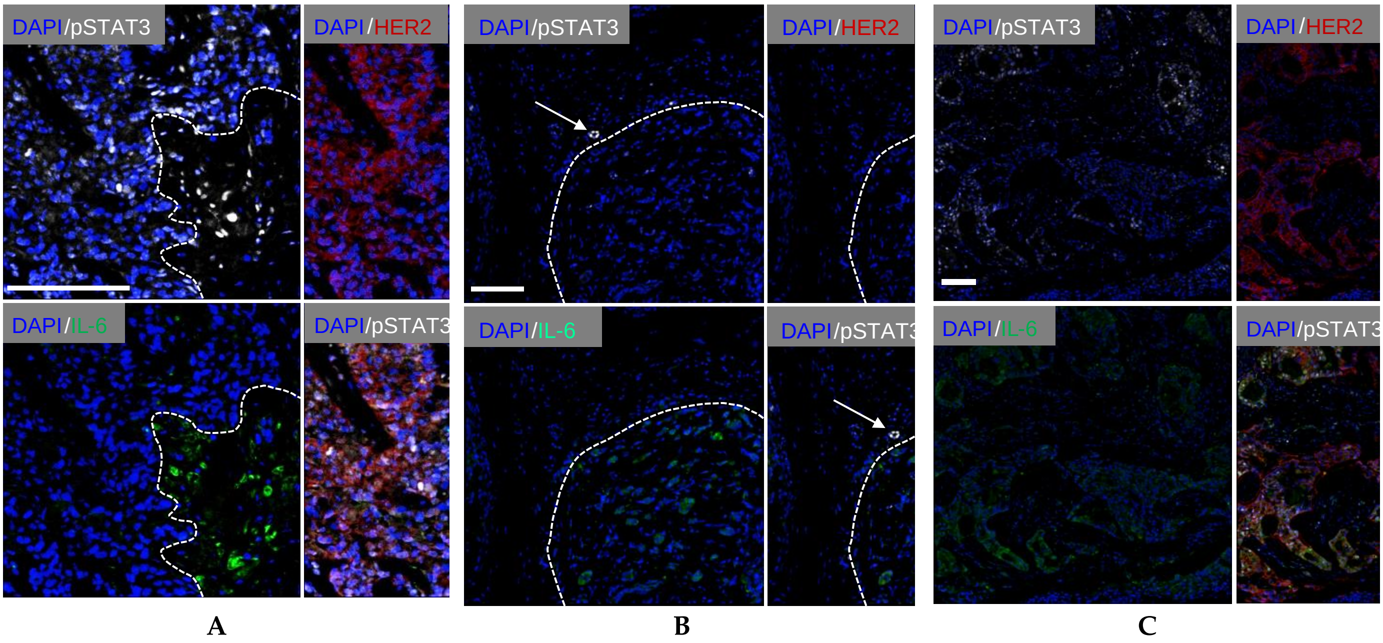

Figure S1. A. Localization of HER2, pSTAT3 and IL-6 in human inflammatory breast cancers. FFPE sections from human inflammatory breast cancer pre-treatment core biopsies were co-stained with pSTAT3 (white), HER2 (red), and IL-6 (green) antibodies using multiplexed multispectral imaging and counterstained with DAPI (blue) to visualize the nuclei (see Materials and Methods). The specimen shown is a luminal (ER^{pos}, PgR^{pos}, $HER2^{neg}$) tumor (INFLAM #4, **Table S1**), which displays co-localization of cell surface HER2 expression and nuclear pSTAT3 in a region of tumor cells and diffuse IL-6 staining in an adjacent region of stroma (**Pattern 2**). Scale bar represents 100 µm. **B.** Localization of HER2, pSTAT3 and IL-6 in human inflammatory breast cancers. FFPE sections from human inflammatory breast cancer pre-treatment core biopsies were co-stained with pSTAT3 (white), HER2 (red), and IL-6 (green) antibodies using multiplexed multispectral imaging and counterstained with DAPI (blue) to visualize the nuclei (see Materials and Methods). The specimen shown is a luminal (ER^{pos}, PgR^{pos}, $HER2^{neg}$) tumor (INFLAM #8, **Table S1**), which displays nuclear pSTAT3 positivity in a small cluster of tumor cells (white arrow) adjacent to an $IL\text{-}6^{pos}/pSTAT3^{neg}$ tumor region (**Pattern 3**). Scale bar represents 100 µm. **C.** Localization of HER2, pSTAT3 and IL-6 in human inflammatory breast cancers. FFPE sections from human inflammatory breast cancer pre-treatment core biopsies were co-stained with pSTAT3 (white), HER2 (red), and IL-6 (green) antibodies using multiplexed multispectral imaging and counterstained with DAPI (blue) to visualize the nuclei (see Materials and Methods). The specimen shown is a HER2 amplified/enriched (ER^{neg}, PgR^{neg}, $HER2^{pos}$) tumor (INFLAM #8, **Table S1**), which displays co-localization of each of cell surface HER2 expression, nuclear pSTAT3, and IL-6 in tumor cells (**Pattern 4**). Scale bar represents 100 µm.

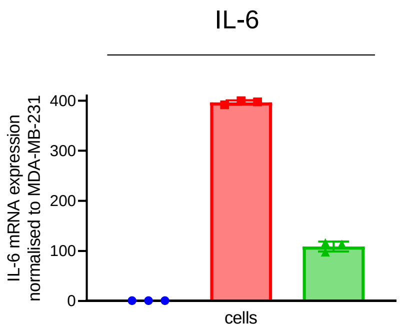

Figure S2. RNA-Seq analysis of IL-6 mRNA expression in cultured TNBC cell lines. RNA-Seq data (Gene Expression Omnibus (GEO) Accession # GSE180788) was interrogated for IL-6 mRNA levels. Expression in MDA-MB-231 non-IBC TNBC cells was compared to A3250 and SUM149 IBC TNBC cells cultured *in vitro* [11]. Expression in MDA-MB-231 was set to 1. Mean ± SD (*n* = 3). IL-6 mRNA levels in A3250 are approximately four fold higher than in SUM149. Data taken from Rogic *et al.*, 2021 [11].

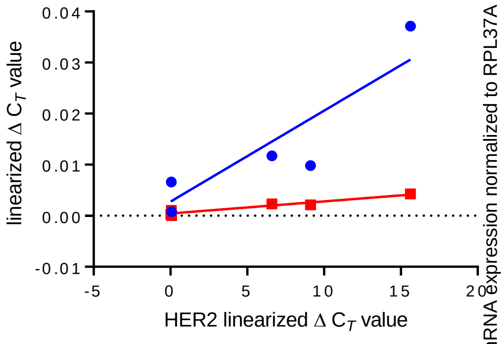

Figure S3. Correlation between IL-6R and HER2 mRNA expression in IBC cell lines. Linearized ΔC_T values from TaqMan qRT-PCR analyses of IL-6R or sIL-6R (y axis) and HER2 (x axis) mRNA expression were taken from Figure 2 and plotted. Simple linear regression analysis carried out using Prism v8 (GraphPad Software, San Diego, CA, USA) showed significant correlation of IL-6R and HER2 mRNA expression (r^2 = 0.80; *, $p < 0.05$), and of sIL-6R and HER2 mRNA expression (r^2 = 0.93; **, $p < 0.01$).

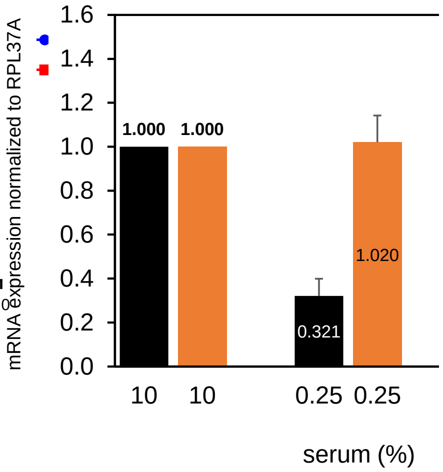

Figure S4. TaqMan qRT-PCR analysis of IL-6 (black) and IL-6R (orange) mRNA expression in SUM190 cells in full serum (10%) and low serum conditions. SUM190 cells were cultured in 10, 0.25, or 0% serum for 48h prior to RNA isolation. Expression was set to 1 in 10% serum condition. Mean ± SD (n = 3). IL-6, p = ***. IL-6R, p = **, by one-way ANOVA.

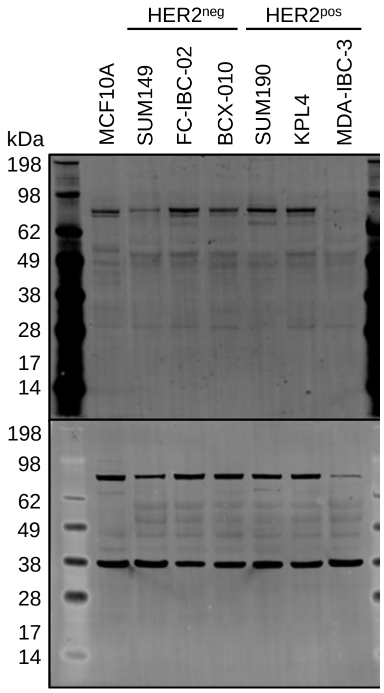

Figure S5A. Original Western blot corresponding to Figure 3A.

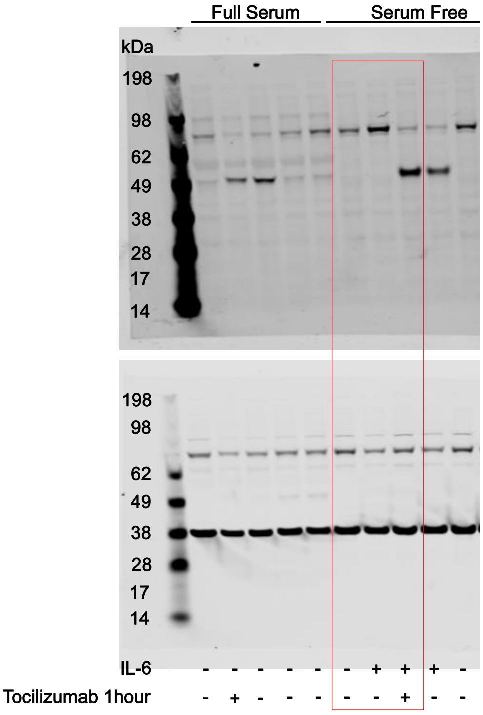

Figure S5B. Original Western blot corresponding to Figure 3C (SUM149).

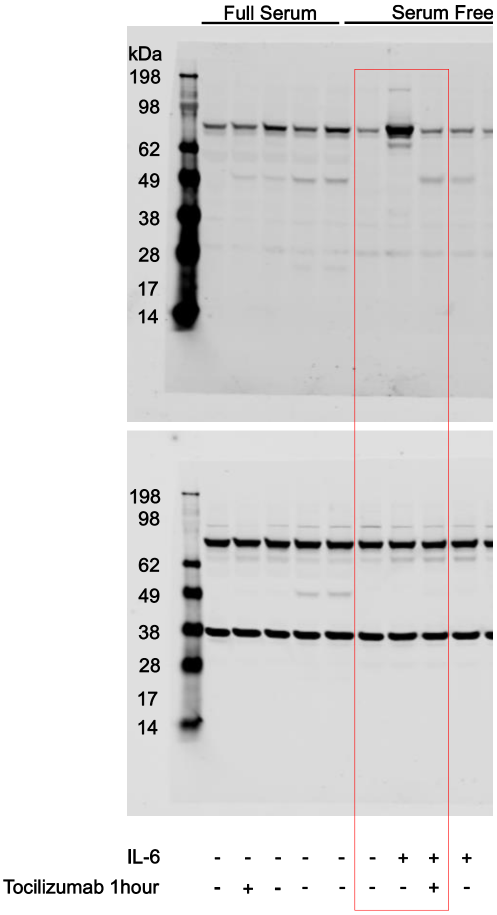

Figure S5C. Original Western blot corresponding to Figure 3C (FC-IBC-02).

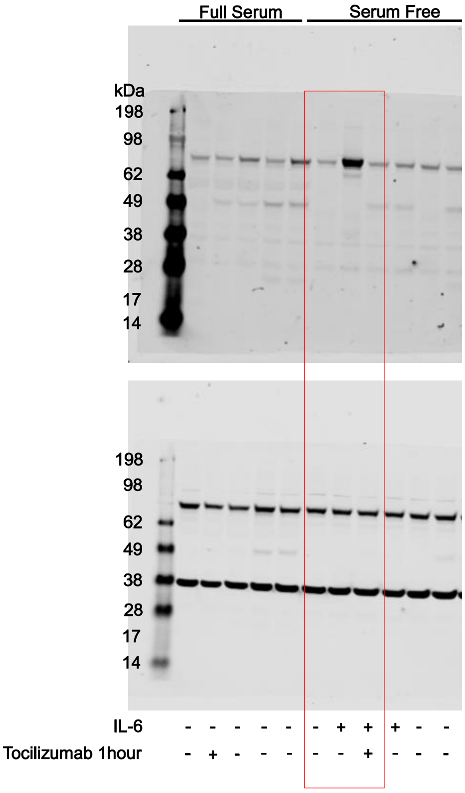

Figure S5D. Original Western blot corresponding to Figure 3C (BCX-010).

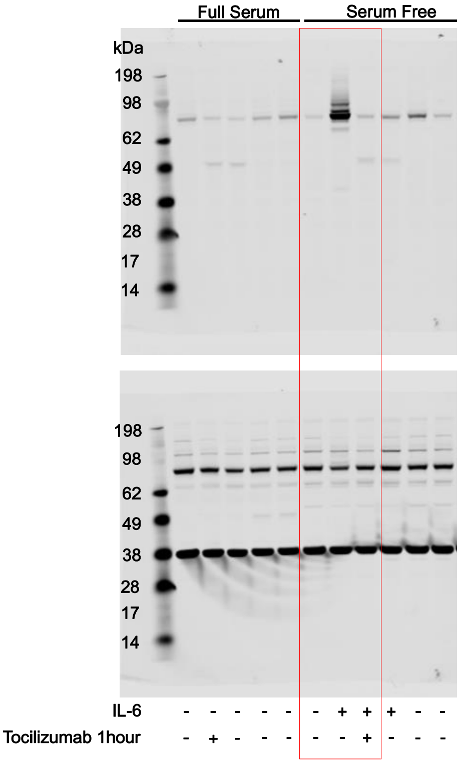

Figure S5E. Original Western blot corresponding to Figure 3C (SUM190).

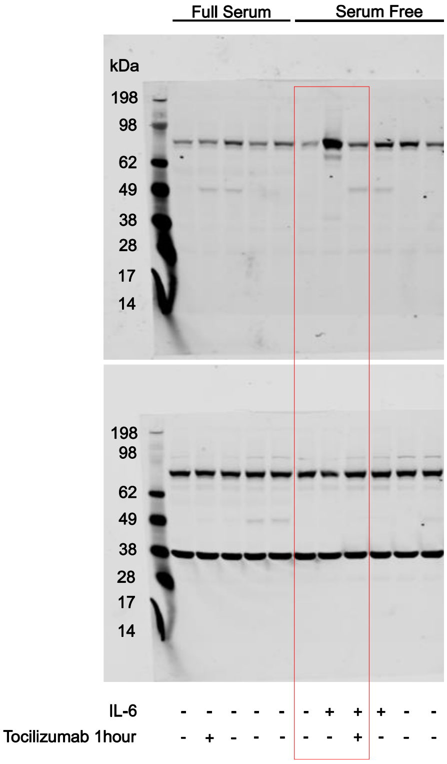

Figure S5F. Original Western blot corresponding to Figure 3C (KPL4).

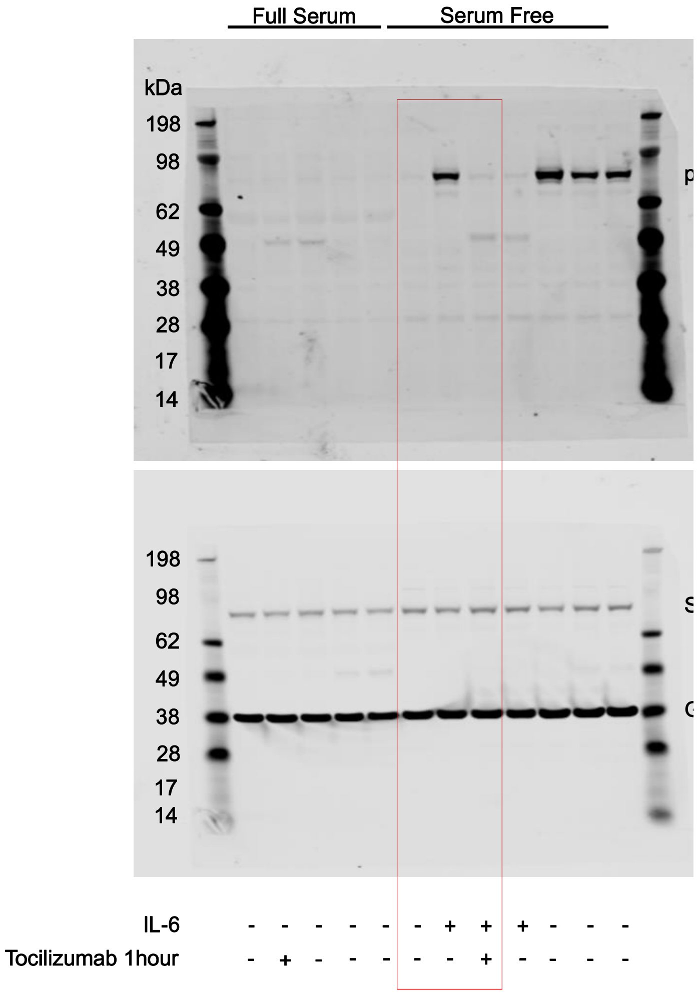

Figure S5G. Original Western blot corresponding to Figure 3C (MDA-IBC-3).

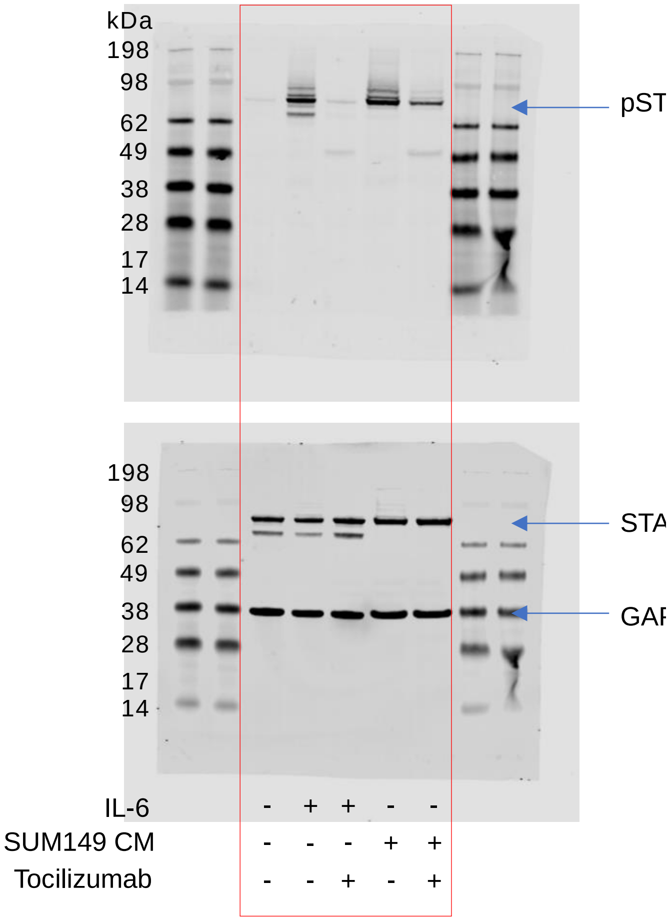

Figure S5H. Original Western blot corresponding to Figure 4A.

Table S1. Clinical variables of the human inflammatory breast cancer cohort. A collection of inflammatory breast cancer specimens was assembled from 18 different patients divided into pre-surgical biopsies (left side) and surgical specimens obtained following neo-adjuvant therapy (right side). Clinical and histopathological variables of both pre-surgical and surgical specimens are indicated. Pre-treatment core biopsies were obtained all 18 patients, 17 of whom proceeded to neo-adjuvant therapy with the treatments indicated. Thirteen of the 17 patients who received neo-adjuvant therapy (76%) proceeded to surgery. Two of 18 (11%) patients achieved a complete pathological response (pCR) to neo-adjuvant therapy. Six of 18 tumors were HER2 gene amplified (total HER2 positive rate of 6/18 = 33%) and four tumors were classified as triple-negative (TNBC, total TNBC rate of 4/18 = 22%). One patients' tumor (INFLAM #26) switched pathological subtype following neo-adjuvant therapy. Fifteen of 18 pre-treatment biopsies (tissue ID's INFLAM #2 - #17) were available for study and were analyzed for HER2, pSTAT3, and IL-6 protein expression (see Figure 1, Figure S1A–C). Patterns of expression that include a $pSTAT3^{pos}/IL\text{-}6^{neg}$ tumor region adjacent to an inversely stained $pSTAT3^{neg}/IL\text{-}6^{pos}$ region (Pattern 1, Pattern 2, Pattern 3) are highlighted in red font.

Legend: AC, Doxorubicin and Cyclophosphamide. AI, Aromatase Inhibitor. Caelyx, liposomal doxorubicin. ER, Estrogen Receptor. FEC-D, Fluorouracil, Epirubicin, Cyclophosphamide, Docetaxel. HER2, Human Epidermal Growth Factor Receptor 2. IDC, Invasive Ductal Carcinoma. ILC, Invasive Lobular Carcinoma. ISH, *In Situ* Hybridization. LTFU, lost to follow up. M, Metastasis. MBC, Metastatic Breast Cancer. N/A, Not Applicable. N, Node. Neg, Negative. pCR, Pathological Complete Response. PgR, Progesterone Receptor. Pos, Positive. T, Tumor. TNBC, Triple Negative Breast Cancer. X, Undetermined.
